# Critical analysis of Chagas disease treatment in different countries

**DOI:** 10.1590/0074-02760210034

**Published:** 2022-07-08

**Authors:** Fernanda de Souza Nogueira Sardinha Mendes, Jose Antonio Perez-Molina, Andrea Angheben, Sheba K Meymandi, Sergio Sosa-Estani, Israel Molina

**Affiliations:** 1 Fundação Oswaldo Cruz-Fiocruz, Instituto Nacional de Infectologia Evandro Chagas, Rio de Janeiro, RJ, Brasil; 2 Ramón y Cajal University Hospital, Instituto Ramón y Cajal de Investigación Sanitaria, Infectious Diseases Department, National Referral Unit for Tropical Diseases, Madrid, Spain; 3 Istituto di Ricovero e Cura a Carattere Scientifico Sacro Cuore Don Calabria Hospital Department of Infectious - Tropical Diseases and Microbiology, Negrar di Valpolicella, Verona, Italy; 4 University of California, Center of Excellence for Chagas Disease at Olive View, Los Angeles, CA, US; 5 Drugs for Neglected Diseases initiative, Rio de Janeiro, RJ, Brasil; 6 National Scientific and Technical Research Council, Epidemiology and Public Health Research Center, Buenos Aires, Argentina; 7 Vall d’Hebron University Hospital, Department of Infectious Diseases, Programa de Salut Internacional de l’Institut Català de la Salut, Barcelona, Spain; 8 Fundação Oswaldo Cruz-Fiocruz, Instituto René Rachou, Laboratório de Triatomíneos e Epidemiologia da Doença de Chagas, Belo Horizonte, MG, Brasil

**Keywords:** Chagas disease, treatment, benznidazole, nifurtimox

## Abstract

As a result of globalization and constant migratory flows, Chagas disease is now present in almost all continents. The management and treatment of the disease is often influenced by the economic and social context of the societies that host patients. In this manuscript, we aim to provide a comparative review of approaches to patients with Chagas disease in the Americas and Europe.

At the beginning of the 20th century, the Brazilian physician Dr Carlos Chagas discovered the flagellated protozoan *Trypanosoma cruzi*, causal agent of American trypanosomiasis or Chagas disease.^1^

Constant migratory movements have made it possible for a disease, initially linked to rural areas of Latin America, to be currently considered a global public health challenge.

A comprehensive and multisectoral approach is needed to control Chagas disease efficiently and effectively. Significant and sustained political will is, therefore, necessary to consider this goal as both feasible and achievable.

Through large collective initiatives, various national programs, scientific societies, and supranational organizations have greatly contributed to the reduction of disease indicators.

Programs such as the control of domestic vector populations and the implementation of screening programs in blood banks and among women of childbearing age, amongst others, have undoubtedly had an impact on progressively reducing transmission of Chagas disease.

Timely identification and antiparasitic treatment of Chagas disease has important benefits, including prevention of future congenital transmission in treated mothers, serological cure in infants and children, and reduction of progression to advanced forms of the disease in adults.^2^ ^,^ ^3^ ^,^ ^4^ ^,^ ^5^ ^,^ ^6^ However, once the disease has progressed to an advanced phase with severe cardiac or digestive disease, etiological treatment does not appear to have clinical benefits.^7^ Therefore, early screening, diagnosis, and antiparasitic treatment of Chagas disease, in addition to representing a benefit for the patient, can also be considered as a public health strategy.

Geography and the socioeconomic and cultural context determine the treatment of patients with Chagas disease. Although access to a correct diagnosis is the main barrier to treatment, other determinants inherent to health systems modify the management of the disease beyond the scientific recommendations.

In this work, we aim to review antiparasitic treatment approaches to patients with Chagas disease, according to their geographic context.

**Current treatment options**

Treatment of Chagas disease still relies on old drugs licensed more than 50 years ago: nifurtimox (NFX, launched by Bayer in 1965) and benznidazole (BNZ, launched by Roche in 1971). These are the only drugs with proven efficacy against *T. cruzi* infections.^8^

Both compounds are considered to be very effective in acute and recent infections, and for the prevention of maternal-fetal transmission. Unfortunately, their cure ratio, determined through serological tests, declines in people with chronic infection, especially those over 18 years of age.^9^ ^,^ ^10^

BNZ is a nitroimidazole derivative that was first described as an anti-trypanocidal drug in the late 1960s.^11^ It is activated by trypanosomal type 1 nitroreductase, releasing other compounds which bond to guanosine bases in DNA and RNA, resulting in its blockade and making the parasite susceptible to oxidative damage in all stages of the *T. cruzi* life cycle.^12^

NFX is a nitrofuran derivative that was first used clinically in 1969.^13^ After being metabolized by nitroreductases, nitroanion radicals are generated, which in the presence of oxygen, produce free radicals. These radicals block DNA synthesis and accelerate its degradation.^14^

Treatment during the acute phase is highly effective. Cure rates between 65 and 80% have been documented, reaching almost 100% in cases of congenital transmission treated during the first years of life. In cases of chronic infection, cure rates using serology are achieved in between 60 and 93% of children aged up to 13 years, and between 2 and 40% in adults with late chronic disease.^8^ Moreover, patients of all ages treated with either drug have a reduction in parasitemia.^15^

Despite the limited rates of cure in the chronic phase, current recommendations advocate treatment for patients in the chronic phase if they do not have severe heart disease.^16^

This consensus is based mainly on the inferior long-term clinical progression observed in patients treated with benznidazole for the prevention of chagasic cardiomyopathy after an average follow-up of about 10 years, as well as in the prevention of congenital transmission of children born to infected and treated women of childbearing age.^8^

Moreover, serological tests are still used to determine the patients cure. Interpreting their results after treatment or the time needed until cure is documented, make that approach a non-practical way. Development of new biomarkers kits are needed to fill that gap to provide evidence of cure timely in short time after treatment.

The main drawback of both drugs is their high adverse event ratio. BNZ is generally preferred over nifurtimox because of its better tolerability profile, but even so, treatment is discontinued in 9-29% of cases, even though adverse reactions are reversible and are severe in less than 1% of cases.^17^

In this context, new compounds have been tested with the aim of improving on the current treatment options. None have shown superiority compared to the old nitro derivatives. The class which has probably been the most extensively evaluated is the nitroimidazoles. Various different clinical trials were developed to evaluate the efficacy and safety of various triazole derivatives, unfortunately, all failed to demonstrate efficacy against *T. cruzi*.^18^ ^,^ ^19^

BNZ is produced by two companies: ELEA-Phoenix, an Argentinian pharmaceutical company registered in Argentina, Bolivia, Chile, Ecuador, El Salvador, Guatemala, Honduras, Nicaragua, Paraguay, the Dominican Republic, and with the Food and Drug Administration (FDA), and prequalified by Pan American Health Organization (PAHO), and Laboratório Farmacêutico de Pernambuco (LAFEPE), a Brazilian public enterprise (registered only in Brazil and prequalified by PAHO). NFX is produced by Bayer, and donated annually to the World Health Organization (WHO)/PAHO strategic fund, and Gador, an Argentinian pharmaceutical company.

In Latin American countries, both the adult and pediatric versions of the ELEA-Phoenix product are approved for use, except in Brazil, where only the LAFEPE BNZ is approved. Despite being registered in most countries in the Americas, BZN and NFX are not routinely available in sufficient quantities at primary healthcare facilities for several reasons, including suboptimal ordering patterns, limited supply/production, and in-country supply chain issues.

In Europe, neither BNZ nor NFX are registered drugs. Nifurtimox is donated by WHO under formal request for a given patient and benznidazole is imported through international distributors and under the authorization of the Ministry of Health of the country in question.

In 2017, BNZ was approved in the USA for use in children with Chagas disease of between 2 and 12 years of age, and it became commercially available on 14 May 2018. Its use for other age groups is off-label. Prescriptions require submission of a completed order form, available at http://www.benznidazoletablets.com/ or by contacting Foundation Care (tel 877-303-7181; fax 877-620-2849; email FastAccess@Exeltis.com). Urgent requests for benznidazole should be made by telephone.^20^ Recently, the FDA also approved NFX for use in pediatric patients (from birth to less than 18 years of age and weighing at least 2.5 kg).^21^ ^)^ Usage for other age groups is off-label, Nifurtimox is available from Bayer distribution centers through commercial pharmacy requests.

**Diagnosis strategies for deciding to treat patients**

There is a consensus on the use of two antigenically different tests such as enzyme-linked immunosorbent assay (ELISA), hemaglutinação indireta (HAI), or imunofluorescência indireta (IFI), although the use of two ELISAs is the more accepted - the first being parasite lysate or crude antigens and the second, recombinant antigens.^16^ IFI is falling into disuse because of commercial shortages, the requirement for specific equipment, and the operator dependent interpretation. In Europe, the chemiluminescent microparticle immune assay (CMIA) has been recently introduced into clinical practice (Spain and Italy) because of its higher accuracy.^22^ In some countries, such as Colombia and Bolivia, tests are performed in series, which is justified by cost-benefit analysis, while in the vast majority of regions, tests are performed in parallel. Generally, for a patient to be considered infected, two positive results must be obtained, although for screening purposes an ELISA or CMIA-based assay may be used as a single test to rule out *T. cruzi* infection.^22^ ^,^ ^23^ ^,^ ^24^

It has been estimated that 2.0-3.3% of serological results are discordant.^25^ In such cases, performing a third test or repeating the serological test 4 to 6 months later resolves discrepancies in more than 50% of cases. For a persistent inconclusive result, a third assay is then indicated to clarify the infection status. While in Latin American countries, IFI, TESA-blot, or western-blot are used to clarify infection status, in Europe or the USA, TESA-blot it is not commercialized.^25^ ^,^ ^26^ Polymerase chain reaction (PCR) techniques are not recommended in these cases because of their low sensitivity. In Europe, an immunoblot has recently become commercially available, but its accuracy as a confirmatory test still needs further investigation (ldbiodiagnostics.com).

Other strategies, such as rapid test or dried-blood spots, have been employed, but mainly in community screening programs and the results are not accepted for initiating anti-trypanocidal treatment.^27^ Some initiatives are evaluating whether a strategy based on the performance of two rapid diagnostic tests might be sufficient to confirm infection and prescribe treatment, mainly in especially vulnerable epidemiological settings.^28^ ^,^ ^29^

**Level of awareness**

In countries and regions considered endemic, management of Chagas disease is recommended at the first level of healthcare, where the care is needed and is provided free of charge. Patients should be evaluated during treatment to monitor possible adverse events. In cases of serious adverse events or progression of the disease, management in specialized centers is recommended. Specialists should evaluate patients with digestive or advanced cardiac manifestations of Chagas disease to indicate specific treatment and regular follow-up.

Conversely, although awareness of this infection in Europe has grown in recent years, most patients are still detected in specialized centers, infectious diseases departments, blood donation facilities, or through specific community-based screening activities.^30^ ^,^ ^31^ ^,^ ^32^ One exception is care for pregnant women, where Chagas disease screening is most widely implemented, although it is not universal.^33^ Although the most vulnerable groups of migrants (children, pregnant women) enjoy exemption from restrictions in many European countries, many barriers to accessing specific health services remain. This is more problematic in insurance-based systems where the registration process may be particularly complicated, as is the case for the USA, where both patients and health providers have limited awareness of the disease.^20^ ^,^ ^34^

**Pretreatment assessment**

Visceral complications should be assessed in the initial evaluation of a patient with chronic Chagas disease. The diagnostic strategy varies according to the clinical history and physical examination. As for asymptomatic patients, a resting electrocardiography (ECG) is performed during the initial examination with or without a chest X-ray, depending on availability. Cardiac ultrasonography is a nonaggressive technique and widely available, ideally all patients with Chagas disease would be candidates for a basal echocardiogram.^35^ In any case, this technique could be optimized for performance in patients with ECG disturbances, men over 30 years, and women over 45.^36^ Additional cardiac tests, such as 24 h ECG Holter monitoring, ergometry, and cardiac MRI, could be considered in symptomatic patients.

Gastrointestinal involvement is not routinely assessed in asymptomatic subjects. Barium swallow and colon enema are the most common diagnostic procedures in symptomatic patients.^37^

**General indications for treatment**

There is a general consensus to treat patients in the acute phase of Chagas disease (regardless of the mechanism of infection), patients with congenital infection, and reactivations in immunosuppressed patients. Patients up to 18 years old and women of child-bearing age are considered the priority target populations. For chronic Chagas disease, treatment is generally offered to subjects in the indeterminate phase, especially those up to 18 years of age, and subjects with mild to moderate disease. Although there is no formal contraindication regarding the upper age limit, it is widely accepted that there is a better risk-benefit balance up to 50 years of age.^16^ In clinical practice, the indication for treatment depends on absence of visceral involvement and the general condition of the patient rather than age. This fact might have an impact on the mean age of patients treated, depending on the country. The mean age of patients receiving treatment could be higher in non-endemic countries than in Latin America for two reasons (i) non-endemic countries (mainly Europe) started to manage patients when the consensus was in favor of treatment and (ii) the vast majority of patients with Chagas disease in non-endemic countries are adults.

Treatment with either BNZ or NFX is not recommended during pregnancy because of their teratogenic potential and a pregnancy test is recommended in women of childbearing age. This measure has not been evaluated from a cost-effectiveness perspective and is, therefore, not widely implemented. Barrier contraception methods or absolute sexual abstinence should be recommended.

**Treatment schemes**

Although there are no randomized clinical trials that compare BNZ and NFX, BNZ is generally preferred due to its better tolerability. Two ongoing studies, TESEO (NCT03981523) and Equity (NCT02369978) will compare the efficacy and tolerability of both compounds.^38^ BNZ is usually administered at 5-7 mg/kg/day, with 2 to 3 daily doses for 60 days (Table).

**TABLE:** Treatment recommendations for Chagas disease

		Benznidazole	Nifurtimox	Grades of recommendation levels of evidence
Acute infections	Congenital	10 mg/kg per day in 2 to 3 daily doses for 60 days	10-15 mg/kg per day in 2 to 3 daily doses for 60 days	AIII
	Vectorial and oral	Children (≤ 40 kg): 7.5-10 mg/kg per day in 2 to 3 daily doses for 60 days	Children (≤ 40 kg): 10-15 mg/kg mg/kg per day in 2 to 3 daily doses for 60 days	AIII
		Adults (> 40 kg): 5-7 mg/kg per day in 2 to 3 daily doses for 60 days	Adults (> 40 kg): 8-10 mg/kg per day in 2 to 3 daily doses for 60 days	AIII
	Laboratory accident	5-7 mg/kg per day in 2 to 3 daily doses for 10-14 days	8-10 mg/kg per day in 2 to 3 daily doses for 10-14 days	AIII
	Post-transfusion or transplant from an infected donor	5-7 mg/kg per day in 2 to 3 daily doses for 60 days	8-10 mg/kg per day in 2 to 3 daily doses for 60 days	AIII
Chronic infections	Immunocompetent patient	Children (≤ 40 kg): 7.5-10 mg/kg per day in 2 to 3 daily doses for 60 days	Children (≤ 40 kg): 10-15 mg/kg mg/kg per day in 2 to 3 daily doses for 60 days	
		Adults (> 40 kg): 5-7 mg/kg per day in 2 to 3 daily doses for 60 days	Adults (> 40 kg): 8-10 mg/kg per day in 2 to 3 daily doses for 60 days	
Special situations	HIV infected patients	Same posology as immunocompetent patient. Primary prophylaxis benznidazole (200mg/ day or 5 mg/kg/day three times a week) until the CD4 lymphocyte count reaches 200-250 cells/mL and viral load is undetectable for at least 6 months in a patient on stable antiretroviral therapy.		BIII
	Reactivation (HIV or transplant recipients)	Benznidazole 5-7.5 mg/kg per day in 2 to 3 daily doses for 60 days	Nifurtimox 8-10 mg/kg per day in 2 to 3 daily doses for 60 - 90 days	AIII
		Higher doses in the case of CNS involvement (15 mg/kg/day). Secondary prophylaxis benznidazole (200mg/ day or 5mg/kg/day three times a week) until the CD4 lymphocyte count reaches 200-250 cells/mL and viral load is undetectable for at least 6 months in a patient on stable antiretroviral therapy (in the case of HIV patients)			

Although the most widely used maximum daily dose is 300 mg, there is published experience of daily doses of 400 mg without a greater adverse event ratio. In those patients who need a daily dose of over 300-400 mg/day, it is recommended to extend the length of treatment up to 80 days rather than to increase the daily dose. There is a general consensus that a course of treatment of at least 60 days is complete. The 30-day option is only followed in Argentina.

Several studies suggest that the use of a simpler fixed dose of BZN may be equivalent to an adjusted dose in terms of effectiveness, which would simplify administration and enhance compliance.^39^ ^,^ ^40^ ^,^ ^41^ A recent meta-analysis did not find any direct evidence to support this hypothesis, but authors suggest that an adjusted dose is probably equivalent in terms of significant safety and efficacy outcomes, while the effect on critical outcomes is uncertain.^42^

**Follow up of patients**

Once treatment is initiated, scheduled visits are important to monitor possible adverse events and antiparasitic treatment compliance. The ideal patient follow-up has not been determined, and schedules differ within and between countries. In general, patients are followed with a blood test and clinical visit 1 to 3 times during the two-month course of treatment (baseline, 30, and 60 days after start of treatment). Many centers in non-endemic areas and countries such as Colombia or Argentina consider one extra visit (in person or by phone) between day 10 and 14, when the most adverse events occur.

Once or twice-yearly follow-up is recommended for patients, both symptomatic and asymptomatic, and irrespective of parasiticidal treatment, with the objective of early detection of clinical progression, implementation of treatment to control visceral complications, and to rule out other risk factors for cardiovascular disease, and potentially promote healthy living habits.^31^ ^,^ ^35^ ^,^ ^43^

*Trypanosoma cruzi* serology and an ECG should be performed annually, while echocardiography may be performed every 2-3 years depending on symptoms or disease severity.

Children must be monitored closely, not only to detect clinical manifestations related to the infection, but to assess cure. Time points for follow-up may vary according to national programs, but it is generally recommended every 6 months in children below 2 years old and annually in children older than 2 years, until two consecutive tests are non-reactive. Persistence of reagent serology or evidence of positive parasitological exams may indicate therapeutic failure, in which case a new course of treatment should be offered.

PCR for *T. cruzi* can also be used to monitor for treatment failure in patients who have been treated. Molecular biology tests are rarely available at the primary health care level in endemic countries, being more accessible in non-endemic countries or in specialized centers.

Retreatment after treatment failure is an unresolved issue worldwide. Failure is most commonly detected via positive PCR in the blood, the frequency of which ranges from 2.3 to 38% during follow-up. In general, patients with treatment failure may be treated again if the indications for therapy remain and no further contraindications have developed. The same or alternative drugs may be used, possibly for long-term treatment (ideally for 90 days, rather than the standard 60-day schedule).^9^ ^,^ ^18^ ^,^ ^44^ ^,^ ^45^ ^,^ ^46^

**Special situations**

Screening programs: pregnant women, blood banks, and organ transplantation.

Chagas disease has arisen as a public health concern in many regions where it is not endemic, and in these areas where vectorial and oral transmission do not occur, otherwise less common routes, such as transmission through blood transfusion, organ transplantation from an infected donor, or from mother to child, are of increasing importance.

In endemic countries, the trend is for countries to gradually adopt the recommendation of universal screening in pregnant women. Bolivia, Argentina, and Uruguay have universal screening regulations throughout their territory. Chile has screening in endemic regions for vector transmission. Brazil has two states with universal screening of pregnant women (Goiás and Mato Grosso do Sul). There are supranational initiatives in progress (MTCT Plus, the elimination of mother-to-child transmission of HIV infection, syphilis, congenital Chagas disease, and perinatal infection with the hepatitis B virus) where the screening of newborns for multiple diseases is recommended. These initiatives are progressively aimed at screening in the region.^47^

In recent years, mandatory clinical-epidemiological and serological screening has been established for blood and tissue donors in most endemic countries. Therefore, a great reduction in the risk of transmission of Chagas disease throughout Latin America has been observed.^48^ ^,^ ^49^

Some European countries, particularly those with many Latin American immigrants, have implemented protocols at different levels to prevent such routes of infection. With reference to transmission through blood transfusion, seven countries have either already implemented, or are in the process of implementing, changes to their recommendations to enhance detection of cases of *T. cruzi* infection: France, Italy, Portugal, Spain, Sweden, Switzerland, and the United Kingdom.^33^

With regard to health policies for solid organ transplantation, only three countries (Italy, Spain, and the United Kingdom) have national guidelines to control this route of transmission through systematic screening of all donors at risk of infection.

In the United States, blood donor screening is universal but only one time for each donor. Subsequent screening will be only performed whether the blood bank identifies them as having altered risk (a prolonged stay in Latin America, for example).^50^

Organ donor screening is only conducted in some organ procurement organizations and is generally risk-based. Screening of donors (blood and organ) has been based largely on risk assessment and generally implemented since 2007.^20^ ^,^ ^50^ ^,^ ^51^

In non-endemic regions, screening for Chagas disease in asymptomatic Latin American adults is highly relevant, since it has been demonstrated that early diagnosis at primary care level and ulterior treatment is a cost-effective strategy.^52^

*HIV co-infection* - Although *T. cruzi* and HIV co-infection is well documented, existing data about symptomatic cases comes from isolated case reports and series published before the extensive use of combined antiretroviral therapy or from patients not taking antiretrovirals. Since the first case was reported in 1990,^53^ the epidemiological trend of Chagas patients moving from rural areas to urban regions, means that coinfection has been described more frequently in the recent years.^54^ In any case, recommendations for treatment and prophylaxis are homogeneous within all regions.

In endemic countries, the coinfection rate ranges from 1.3 to 7.1% and is slightly higher in intravenous drug users (8.9%),^55^ ^,^ ^56^ ^,^ ^57^ reflecting the possibility of transmission through shared syringes with infected blood.

In HIV-infected patients, *T. cruzi* can behave as an opportunistic parasite with reactivation the most life-threatening complication. Reactivation typically occurs with a CD4 count less than 200 cells per μL, and mainly when it is less than 100 cells per μL. Rates of this complication can be as high as 15 to 35% in patients not taking antiretroviral treatment. The most common clinical manifestations are meningoencephalitis, cerebral chagoma, acute myocarditis, and panniculitis.^58^

As for asymptomatic co-infected individuals, the same parasiticidal treatment schedule is recommended. In cases of reactivation, prompt parasiticidal treatment and at higher doses in the case of CNS involvement (15 mg/kg/day) should be initiated. A treatment duration of longer than 60 days may be needed. Although there is scarce information on the effect of antiretroviral treatment in reactivations, it does not seem to increase the risk of immune reconstitution inflammatory syndrome, thus early use of antiretrovirals is highly recommended. Secondary prophylaxis after reactivation has occurred is recommended, especially during CD4 cell recovery and HIV-viral load control. BNZ can be used (200 mg/ day or 5 mg/kg/day three times a week) until the CD4 lymphocyte count reaches 200-250 cells/mL and the viral load is undetectable for at least 6 months in a patient on stable antiretroviral therapy.^55^ ^,^ ^58^ ^,^ ^59^

Therefore, considering the risk of reactivation and its bad prognosis, screening for *T. cruzi* should be performed in all HIV-infected individuals, individuals potentially exposed to *T. cruzi* infection, and in the children of HIV infected mothers.

**Access to treatment**

Access to both BNZ and NFX presents a challenge in most non-endemic countries, as well as for patients living in poor rural areas of endemic countries, due, among other causes, to restricted access to health care systems and limited provider awareness.

In endemic areas, treatment coverage is extremely low, reaching only about 1% of estimated cases.

Even though BNZ was recently approved by the FDA, patients with Chagas disease still have difficulty getting medication.^20^

In addition, in Latin America, there is a significant gap between national demand for etiologic treatment and estimates of the prevalence of the disease. This undoubtedly highlights the long road that remains to be traveled in the diagnosis and treatment of Chagas disease worldwide (Figs 1 and 2).

**Fig. 1:**
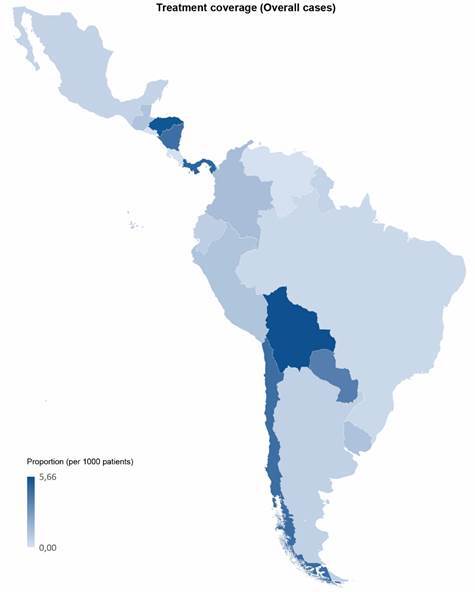
treatment coverage per overall cases. Colors represent the ratio between the average of treatments prescribed (benznidazole plus nifurtimox) between 2017 and 2019 and the estimated number of patients per country. Numbers are expressed by treatments per 1000 patients. Data related to treatment have been obtained from PAHO and Laboratorio Elea (benznidazole manufacturer). Data related to patients have been extracted from Chagas disease in Latin America: an epidemiological update based on 2010 estimates.^63^

**Fig. 2:**
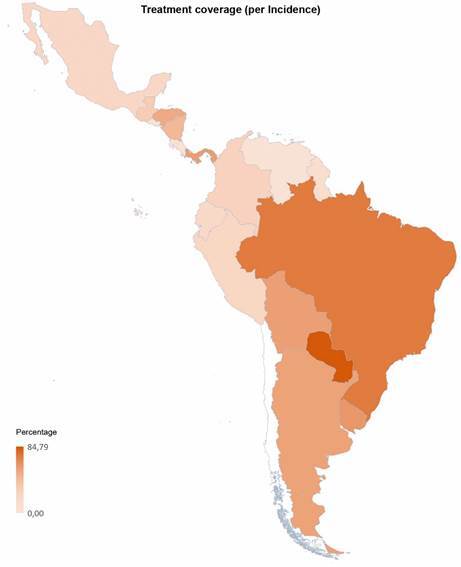
treatment coverage per incidence. Colors represent the ratio between the average of treatments prescribed (benznidazole plus nifurtimox) between 2017 and 2019 and the estimated new cases per year per country (estimated annual number of new cases due to vectorial transmission plus estimated cases due to congenital transmission). Numbers are expressed by percentages. Data related to treatment have been obtained from PAHO and Laboratorio Elea (Benznidazole manufacturer). Chile has been not included in the figure because the proportion of treatments compared to the annual incidences yields a percentage of 466%. Data related to patients have been extracted from Chagas disease in Latin America: an epidemiological update based on 2010 estimates.^63^

Several initiatives are looking for feasible strategies to reduce the barriers to access to diagnosis and treatment. Recently an article described three collaborative projects focused on increasing access to testing and treatment for CD through primary healthcare facilities in Bolivia, Argentina, and Colombia.^60^ In addition, adoption of mandatory notification of chronic cases is fundamental for surveillance systems to be able to highlight the real burden of the disease.^61^ ^,^ ^62^

**Future perspectives**

Future therapeutic options against Chagas disease will be characterized by new therapeutic regimens for the old drugs, BNZ and NFX.

Despite recent discoveries about the biology of the parasite, as well as advances in the drug-discovery process and accessing compound libraries, only a few molecules have been tested in clinical trials and even fewer will reach the market.

Because the major drawback of nitroderivative-based therapy is its toxicity, which hampers its efficacy rate, different approaches have been designed to improve its tolerability.

Several initiatives are evaluating simplified schemes of these compounds. The first study to obtain results, the BENDITA study,^39^ showed that a regimen of BNZ 300 mg/day for two weeks of treatment had the same rate of therapeutic failure compared to the control arm (BNZ 300 mg/day for 8 weeks). The intention-to-treat primary efficacy analysis showed that 82.8% of patients had sustained parasitemia clearance at 12 months on BNZ 300 mg for 8 weeks compared to 79.3% with the two-week regimen.

Although these new strategies may not improve efficacy, they may represent a great advance in terms of public health by improving the safety profile and compliance of patients and healthcare providers at a lower cost.

Reducing the current treatment period from 8 to 2 weeks would greatly facilitate adherence for patients. Currently, patients often prefer to forego treatment due to the long duration and the side effects involved, which can imply lost time from work, difficulty in managing household activities such as care for children, and an inability to participate in community life. Moreover, returning to the clinic for laboratory monitoring during the treatment period can represent additional costs and lost time for patients, many of whom must pay out of pocket for travel, food, and accommodation to reach the nearest available clinic.

From a healthcare provider standpoint, a 2-week treatment period will greatly facilitate the process by reducing the number of patient visits, the amount of monitoring required, and the frequency of side effects requiring additional management. This in turn should reduce the cost of treatment for health systems (although it should be noted that treatment is highly cost effective even with the current standard regimen). Moreover, training of healthcare personnel in administering treatment would be simplified. A simplified treatment would be a powerful tool, enabling scale up of treatment coverage at the level needed to control Chagas disease and eliminate congenital transmission.

Phase III trials are being designed to reinforce the level of evidence, with the intention of carrying them out in 2021.
